# Differential Contribution of the First Two Enzymes of the MEP Pathway to the Supply of Metabolic Precursors for Carotenoid and Chlorophyll Biosynthesis in Carrot (*Daucus carota*)

**DOI:** 10.3389/fpls.2016.01344

**Published:** 2016-08-31

**Authors:** Kevin Simpson, Luis F. Quiroz, Manuel Rodriguez-Concepción, Claudia R. Stange

**Affiliations:** ^1^Plant Molecular Biology Laboratory, Department of Biology, Faculty of Sciences, University of ChileSantiago, Chile; ^2^Centre for Research in Agricultural Genomics, Consejo Superior de Investigaciones Científicas–Institut de Recerca i Tecnologia Agroalimentàries–Universitat Autònoma de Barcelona–Universitat de BarcelonBarcelona, Spain

**Keywords:** carrot, storage root, deoxyxylulose 5-phosphate synthase (DXS), deoxyxylulose 5-phosphate reductoisomerase (DXR), carotenoids, chlorophylls

## Abstract

Carotenoids and chlorophylls are photosynthetic pigments synthesized in plastids from metabolic precursors provided by the methylerythritol 4-phosphate (MEP) pathway. The first two steps in the MEP pathway are catalyzed by the deoxyxylulose 5-phosphate synthase (DXS) and reductoisomerase (DXR) enzymes. While DXS has been recently shown to be the main flux-controlling step of the MEP pathway, both DXS and DXR enzymes have been proven to be able to promote an increase in MEP-derived products when overproduced in diverse plant systems. Carrot (*Daucus carota*) produces photosynthetic pigments (carotenoids and chlorophylls) in leaves and in light-exposed roots, whereas only carotenoids (mainly α- and β-carotene) accumulate in the storage root in darkness. To evaluate whether DXS and DXR activities influence the production of carotenoids and chlorophylls in carrot leaves and roots, the corresponding *Arabidopsis thaliana* genes were constitutively expressed in transgenic carrot plants. Our results suggest that DXS is limiting for the production of both carotenoids and chlorophylls in roots and leaves, whereas the regulatory role of DXR appeared to be minor. Interestingly, increased levels of DXS (but not of DXR) resulted in higher transcript abundance of endogenous carrot genes encoding phytoene synthase, the main rate-determining enzyme of the carotenoid pathway. These results support a central role for DXS on modulating the production of MEP-derived precursors to synthesize carotenoids and chlorophylls in carrot, confirming the pivotal relevance of this enzyme to engineer healthier, carotenoid-enriched products.

## Introduction

Many isoprenoids are present in plants and some of them act as primary metabolites with roles in respiration, photosynthesis, and regulation of growth and development. In plastids, the common precursors of all isoprenoid products, the 5-carbon units isopentenyl diphosphate (IPP) and dimethylallyl diphosphate (DMAPP), are produced by the methylerythritol 4-phosphate (MEP) pathway. MEP-derived precursors are used for the synthesis of isoprenoids such as volatiles (monoterpenes, diterpenes, isoprene), hormones (gibberellins, cytokinins, abscisic acid, strigolactones), and photosynthesis-related compounds (carotenoids, chlorophylls, tocopherols, and prenylquinones). Carotenoids are also responsible for the yellow, orange, and red color of non-photosynthetic organs like flowers and fruits, participating in the attraction of pollinators and seed dispersing agents (Grotewold, 2006).

**FIGURE 1:**
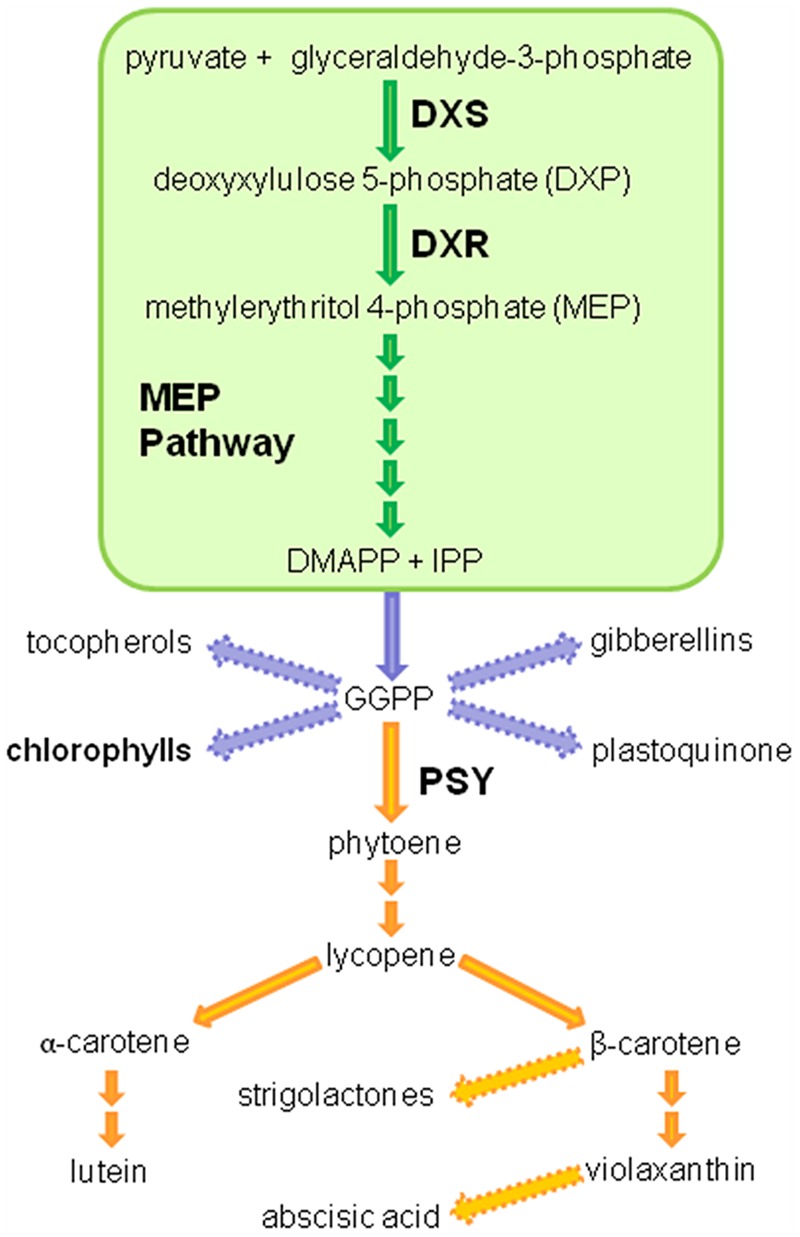
**Isoprenoid and carotenoid pathway in plants.** A simplified schematic representation of the plastidial methylerythritol 4-phosphate (MEP; 2-*C*-methyl-D-erythritol-4-P) and carotenoid pathways. DXS, deoxyxylulose 5-phosphate synthase; DXR, deoxyxylulose 5-phosphate reductoisomerase; IPP, isopentenyl pyrophosphate; DMAPP, dimethylallyl pyrophosphate; GGPP, geranylgeranyl pyrophosphate; PSY, phytoene synthase.

The first step in the MEP pathway, which is catalyzed by the deoxyxylulose 5-phosphate (DXP) synthase (DXS) enzyme, is the formation of DXP from pyruvate and glyceraldehyde 3-phosphate. In the second reaction of MEP pathway the DXP reductoisomerase (DXR) enzyme synthetizes MEP by an intramolecular rearrangement and reduction of DXP. Then, five more enzymes convert MEP (the first intermediate that is specific of this pathway) into IPP and DMAPP (**Figure 1**) (Rodriguez-Concepcion and Boronat, 2002). Different studies have shown that DXS has a major role in the control of the MEP pathway flux (Rodriguez-Concepcion and Boronat, 2015). A recent work actually showed that DXS displays the highest flux control coefficient of the pathway, i.e., it is the main rate-determining enzyme (Wright et al., 2014). In agreement, overexpression of DXS-encoding genes in different plants typically results in increased levels of plastidial isoprenoids such as carotenoids and chlorophylls (Estevez et al., 2001; Enfissi et al., 2005; Carretero-Paulet et al., 2006; Morris et al., 2006; Munoz-Bertomeu et al., 2006; Zhang et al., 2009; Henriquez et al., 2016). Overexpression of DXR-encoding genes also led to increased levels of MEP-derived isoprenoids in many cases (Mahmoud and Croteau, 2001; Carretero-Paulet et al., 2006; Hasunuma et al., 2008; Yang et al., 2012; Chang et al., 2014; Zhang et al., 2015) but had no effect in others (Mendoza-Poudereux et al., 2014).

Like the vast majority of plants, carrot (*Daucus carota*) produces MEP-derived photosynthetic pigments (chlorophylls and carotenoids) in leaves. Both isoprenoids also accumulate at high levels in the root when this organ is exposed to light (Stange et al., 2008; Fuentes et al., 2012; Rodriguez-Concepcion and Stange, 2013). In the dark, however, the storage root of carrot plants only produces carotenoids but at concentrations that are unique among plants. At present, diverse carrot varieties with different carotenoid composition in the storage root exist (Surles et al., 2004; Rodriguez-Concepcion and Stange, 2013; Stange and Rodriguez-Concepcion, 2015). Orange carrots, the most consumed cultivars, get their coloration due to a massive accumulation of carotenoids, principally α- and β-carotene (Surles et al., 2004). The synthesis of carotenoids begins with the addition of three IPP molecules to one DMAPP molecule to produce C20 geranylgeranyl diphosphate (GGPP) by the enzyme GGPP synthase. The condensation of two GGPP molecules catalyzed by the phytoene synthase (PSY) enzyme leads to the production of C40 phytoene in the first committed step of the carotenoid biosynthetic pathway (**Figure 1**). Although it is widely accepted that the production of phytoene represents the first and main rate-limiting step in the biosynthesis of carotenoids (Fraser et al., 2002; Lu and Li, 2008; Maass et al., 2009; Rodriguez-Villalon et al., 2009a; Ruiz-Sola and Rodriguez-Concepcion, 2012), work in model systems such as *Arabidopsis thaliana* and tomato (*Solanum lycopersicum*) has led to propose that the limiting nature of PSY activity largely depends on the availability of the metabolic precursors synthesized in the MEP pathway (Lois et al., 2000; Enfissi et al., 2005; Carretero-Paulet et al., 2006; Rodriguez-Villalon et al., 2009a).

While DXR is often encoded by a single gene in plants, DXS is typically encoded by small gene families with members of at least two functionally specialized classes (Walter et al., 2002; Cordoba et al., 2009; Rodriguez-Concepcion and Boronat, 2015). Type I DXS enzymes supply the precursors for housekeeping and photosynthetic isoprenoids, including chlorophylls and carotenoids, whereas type II isoforms are usually specialized in the production of secondary isoprenoids. A third type of DXS-like sequences is usually present in plant genomes but their enzymatic role is still unclear (Rodriguez-Concepcion and Boronat, 2015). In carrot, one gene for DXR (DCAR_026133) and four DXS-encoding genes (type I DCAR_030576, type II DCAR_009911 and DCAR_014178, and type III DCAR_022887) were recently annotated (Iorizzo et al., 2016). Interestingly, only the expression of the type I DXS gene was correlated with high carotenoid content (Iorizzo et al., 2016). In this work, we aimed to experimentally evaluate the role of DXS and DXR activities in regulating the production of MEP-derived isoprenoids in leaves and storage roots. Because carrot genes encoding these enzymes have only recently become available (Iorizzo et al., 2016), we used the corresponding *Arabidopsis DXS/CLA1* (type I) and *DXR* genes cloned in plant expression vectors under the control of the constitutive *35S* promoter (Pulido et al., 2013; Perello et al., 2016). Analysis of the generated transgenic carrot lines confirmed a limiting role for DXS in the production of both carotenoids and chlorophylls in leaves and roots, whereas DXR appeared to only marginally affect the production of these plastidial isoprenoids in leaves. Most interestingly, DXS overexpression led to increased levels of PSY-encoding transcripts, highlighting the central role of these two enzymes for the control of carotenoid biosynthesis in plants.

## Materials and Methods

### Plant Material

Seeds of commercially acquired carrot *(Daucus carota L.)* cultivar Nantaise were surface sterilized in a solution of 95% ethanol for 1 min and washed once with sterile water for 3 min. Then, the seeds were incubated under agitation in a solution of sodium hypochlorite (2.62% v/v) for 45 min, washed three times with sterile water and finally dried on sterile absorbent paper. The sterile seeds were deposited in sterile flasks with solid MS medium (Murashige and Skoog, 1962) supplemented with 0.44% vitamins, 2% sucrose, 0.01% myo-inositol, 0.7% Agar and pH adjusted to 5.8. The seeds were kept in a growth chamber 3 weeks with a 16 h long day photoperiod illuminated with cool-white fluorescent light (115 μmol m^-2^ s^-1^) at 22°C. Hypocotyls and stems of 3-week-old *in vitro* wild-type carrot plantlets were utilized in *Agrobacterium*-mediated transformation experiments. Transformed carrots were transferred to pots (20 × 10) and cultivated in the greenhouse, as described above.

### *Agrobacterium tumefaciens*-Mediated Transformation of *Daucus carota*

Binary vectors for constitutive expression of *Arabidopsis* genes encoding DXS or DXR proteins fused to GFP were previously reported (Pulido et al., 2013; Perello et al., 2016). *Agrobacterium tumefaciens* (strain GV3101) cells were transformed with these vectors and used for *D. carota* transformation following the protocol described by (Chen and Punja, 2002). Briefly, hypocotyl segments of 3 weeks-old seedlings were co-cultivated with *Agrobacterium* carrying the vector of interest, and placed on solidified MS media (4.4 g/L MS salts, 20 g/L sucrose and 0.7% agar) in darkness. After 2 days, the explants were transferred to solid MS medium containing 1 mg/L 2.4D for somatic embryogenesis induction and supplemented with 0.5 mg/L Basta^®^ and 300 mg/L cefotaxime. After 4 weeks in darkness, the explants were placed on solidified MS medium containing 0.5 mg/L 2.4D, 1 mg/L Basta^®^ and 300 mg/L cefotaxime in photoperiod conditions (16 h light, 115 μmol/m^2^/s). Herbicide-resistant embryos were transferred to MS media in the absence of hormones to induce the development of shoots. After 6 months, transformed plantlets were transferred to soil in a temperature and photoperiod controlled greenhouse (16 h light, 115 μmol/m^2^/s). Different stages of the procedure are shown in Supplementary Figure S1. We obtained several lines per construct and PCR analyses to confirm the presence of the transgenes in the transgenic lines were performed with primers *AtDXRF, AtDXSF*, and *eGFPR* (Supplementary Figure S2; Supplementary Table S1). Transgenic lines developed normally and were visually undistinguishable from non-transgenic controls (Supplementary Figure S3). Four of the lines confirmed to contain the corresponding transgene by PCR (Supplementary Figures S2 and S3) were then picked for quantification of transcript and pigment levels. From those, three representative lines per construct were selected for more detailed analysis.

### Pigment Extraction and High Performance Liquid Chromatography (HPLC) Analysis

Photosynthetic pigments from leaves and roots of wild-type and transgenic plants transferred to soil and grown in the greenhouse for 6 months were extracted from 100 mg of fresh weight with 1 ml of hexane/acetone/ethanol (2:1:1 v/v) as described (Fuentes et al., 2012). The extract was dried with N_2_. To quantify the concentration of chlorophyll *a*, chlorophyll *b* and total carotenoids present in the pigment extracts of leaves and roots of *D. carota*, the pigments extracted were resuspended in 2 mL of acetone and using a spectrophotometer the absorbance was measured at 750, 662, 645, and 470 nm in quartz cuvettes. Absorbance at 662, 645, and 470 nm is used to determine the concentration of chlorophyll *a*, chlorophyll *b* and total carotenoids, respectively. Further, the absorbance at 750 nm was measured to determine the turbidity of the sample because the turbid samples may result in underestimation of the concentration of the pigments concentration. With absorbance measurements, the concentration of chlorophyll *a*, chlorophyll *b* and total carotenoids determined by the equations described (Lichtenthaler and Buschmann, 2001). For α-carotene and β-carotene measurements, the pigments were separated by a HPLC using a RP-18 Lichrocart125-4 reverse phase column (Merck^®^), utilizing a acetonitrile: methanol: isopropanol (85:10:5 v/v) mix as a mobile phase with a 1 ml/min flow rate at room temperature in isocratic conditions. The elution spectra of each maximum were obtained using a diode array detector. The carotenoids were identified according to their absorption spectra, retention time and comparison with specific pigment standards, which was corroborated by comparison with the Carotenoids Handbook (Britton, 1995; Britton et al., 2004). All operations were carried out in triplicate, on ice and dark conditions to avoid photodegradation, isomerization and structural changes of carotenoids.

### RNA Extraction and Quantitative RT-PCR

A frozen powder of 100 mg of *D. carota* leaves from plants transferred to soil and grown in the greenhouse for 6 months was used for total RNA extraction using TRIzol^®^ reagent (Invitrogen) and following the manufacturer’s instructions. For cDNA synthesis, 2 μg of total DNA-free RNA was mixed with 1mM of oligodT primer and Impron II reverse transcriptase (Promega). The expression of the *DXS* and *DXR* transgenes was estimated by RT-PCR using primers *qDXRF, qDXSF* and *qeGFP R* (Supplementary Table S1). Quantitative RT-PCR (qRT) experiments were performed in a Stratagene Mx3000P thermocycler, using SYBR Green double strand DNA binding dye as described previously (Stange et al., 2008). Specific primers for carrot genes were designed targeting the 5′ UTR of *PSY1* (AB032797) and *PSY2* (DQ192187) and the coding sequence of the *18S* gene, selected as the normalizer (Supplementary Table S1). Final data were obtained introducing fluorescence results in the equation described by Pfaﬄ (2001). Each qRT-PCR reaction was performed with three biological replicates and each sample was analyzed in duplicate (technical replicate). In all cases, the reaction specificities were tested with melting gradient dissociation curves and electrophoresis gels. To test for significant differences in gene expression, results were analyzed using the General Linear Models option in the statistical software package Graphpad Prism. The one and two tailed Student *t*-test (*p* < 0.05, confidence interval 95%), were used.

## Results

### The Constitutive Expression of *DXS* Increases Carotenoid Levels in *D. carota* Roots

**FIGURE 2:**
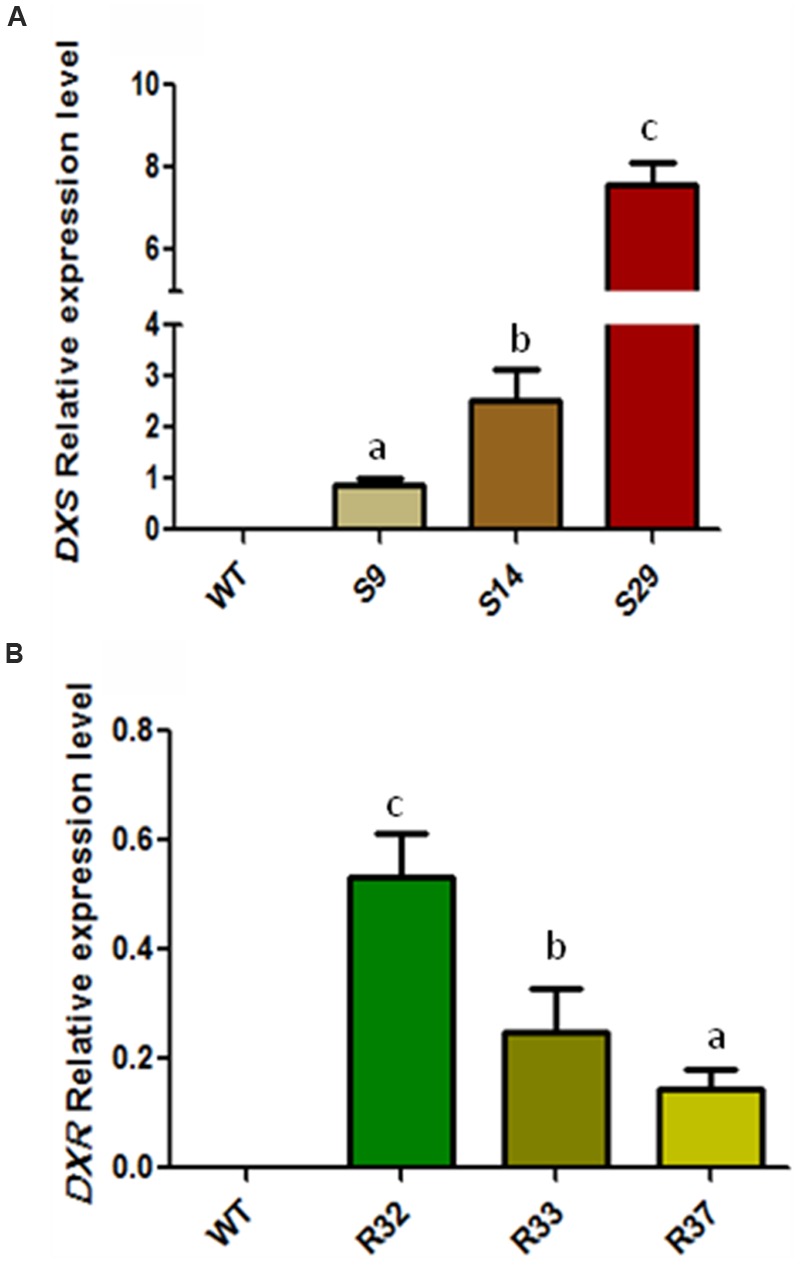
**Relative transcript expression of *AtDXS* and *AtDXR* in leaves of transgenic lines.** Relative transcript abundance of **(A)** *AtDXS* and **(B)** *AtDXR* in representative transgenic carrot lines determined by means of semi quantitative RT-PCR. Assay was carried out in triplicate and normalized to *RNAr18S* expression. Letters indicate significant differences between transgenic lines determined by one-tailed ANOVA and Tukey post-test, *p* < 0.05.

**FIGURE 3:**
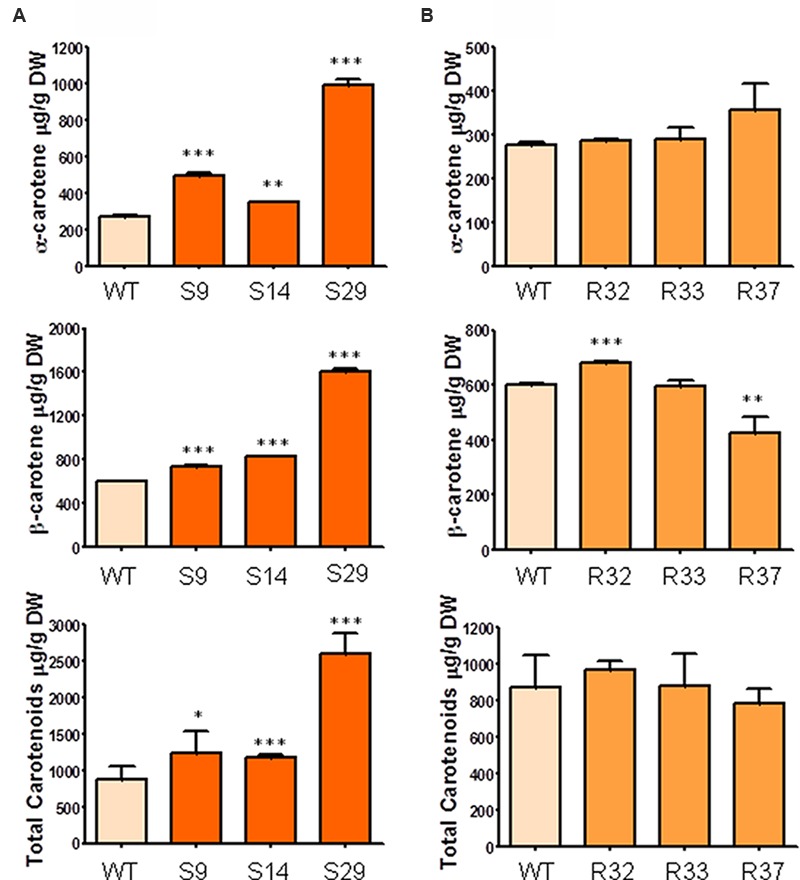
**Carotenoid composition in storage roots of carrot transgenic lines expressing *AtDXS* and *AtDXR*. (A)** Quantification of α-carotene, β-carotene and total carotenoids in storage roots of three DXS transgenic lines, **(B)** Quantification of α-carotene, β-carotene and total carotenoids in the storage root of three DXR transgenic lines. Asterisks indicate significant differences between transgenic lines and wt determined two-tailed unpaired Student’s *t*-test. ^∗^*p* < 0.05; ^∗∗^*p* < 0.01; ^∗∗∗^*p* < 0.001.

In order to determine the significance of the supply of MEP-derived metabolic precursors for the synthesis of carotenoids (and chlorophylls) in carrot, we generated transgenic plants expressing GFP-tagged versions of the *Arabidopsis DXS/DXS1/CLA1* (At4g15560) or *DXR* (At5g62790) genes under the control of the constitutive *35S* promoter (Supplementary Figures S1 and S2). The presence of the corresponding transgene in the generated S (*35S:DXS*) and R (*35S:DXR*) lines was confirmed by PCR (Supplementary Figure S2). After semi-quantitative RT-PCR analyses to estimate transgene expression levels in 6-month-old carrot plants, three representative lines of each construct were selected for further experiments (**Figure 2**). First, we aimed to quantify the accumulation of carotenoids in the root of transgenic plants and untransformed wild-type (WT) controls. As shown in **Figure 3A**, the constitutive expression of *DXS* produced a substantial increment in α-carotene (up to 3.6-fold) and β-carotene (up to 2.7-fold) levels in the storage root. As a consequence, total carotenoids were also significantly higher than in untransformed controls, reaching 2600 μg/g DW (dry weight) in line S29, the one also showing the highest levels of transgene expression (**Figure 2**). On the other hand, the constitutive expression of *DXR* only led to a slight increase in β-carotene in line R32 (the one with highest transgene expression levels) but no significant changes in the levels of α-carotene and total carotenoids in the root (**Figure 3B**). When calculating the mean of all transgenic lines together, the constitutive expression of the *DXS* gene resulted in an average increase of 114% of α-carotene, 75% of β-carotene, and 78% of total carotenoids with respect to WT plants, whereas *DXR* overexpression had no impact on root carotenoid levels (**Figure 3**). These results suggest that DXS, but not DXR, plays an important role in controlling the flow of metabolic precursors toward carotenoid synthesis in the storage root of *D. carota* plants.

### DXS and, to a Lower Extent, DXR Can Influence Carotenoid and Chlorophyll Content in *D. carota* Leaves

**FIGURE 4:**
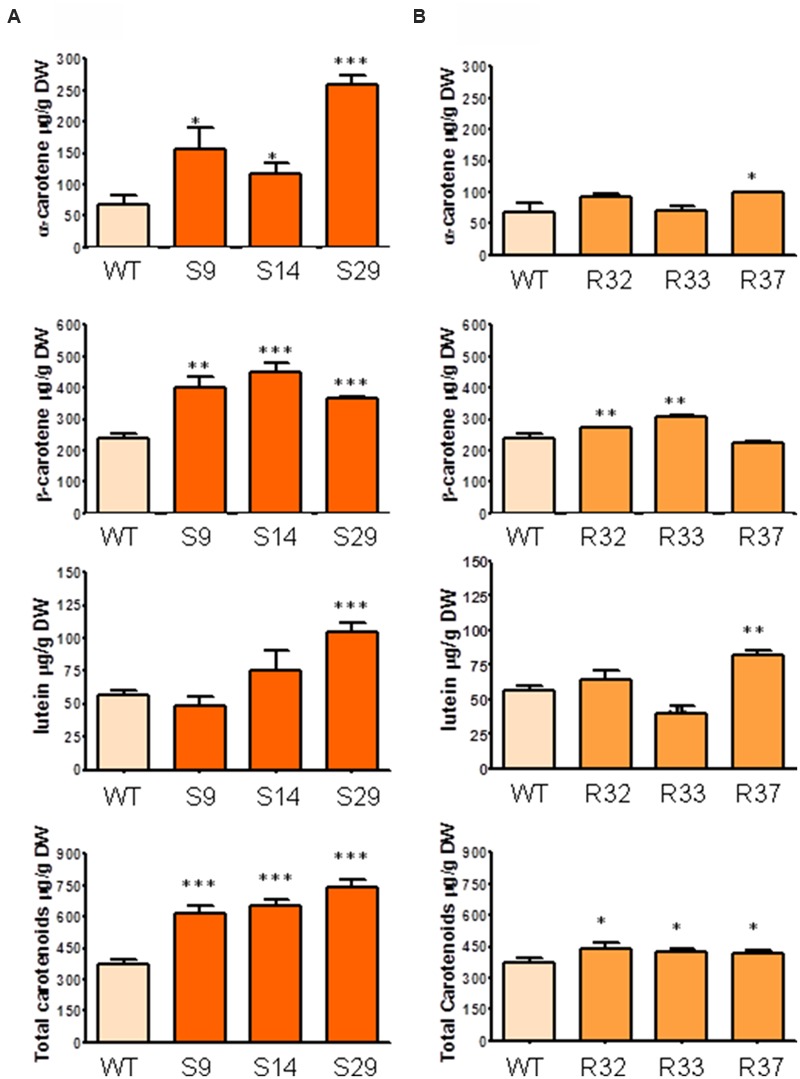
**Carotenoid composition in leaves of carrot transgenic lines expressing *AtDXS* and *AtDXR*. (A)** Quantification of α-carotene, β-carotene, lutein and total carotenoids in leaves of three DXS transgenic lines, **(B)** Quantification of α-carotene, β-carotene, lutein and total carotenoids in leaves of three DXR transgenic lines., Asterisks indicate significant differences between transgenic lines and wt determined by two-tailed unpaired Student’s *t*-test. ^∗^*p* < 0.05; ^∗∗^*p* < 0.01; ^∗∗∗^*p* < 0.001.

We next asked whether the upregulation of DXS or DXR levels could have a different effect on photosynthetic tissues of carrot plants. To address this question, we quantified the levels of both carotenoids and chlorophylls in leaves of the selected transgenic lines. As shown in **Figure 4A**, the constitutive overexpression of *DXS* produced a significant raise in the concentration of total carotenoids. In particular, α- and β-carotene increased in leaves of all lines tested, reaching levels that were up to fourfold and twofold higher, respectively, than those found in untransformed controls. Levels of lutein, however, were only significantly higher in line S29 (**Figure 4A**). On the other hand, the overexpression of *DXR* produced modest but statistically significant increments in α-carotene, β-carotene and lutein in leaves of some of the transgenic lines tested, giving rise to a slight increment in total carotenoids in all transgenic lines (**Figure 4B**). On average, the expression of the *Arabidopsis DXS* gene in *D. carota* produced an increase of 91, 69, 30, and 80% in the concentration of α-carotene, β-carotene, lutein and total carotenoids, respectively, while increasing DXR levels only led to statistically significant increments in lutein (17%) and total carotenoids (19%; **Figure 4**).

**FIGURE 5:**
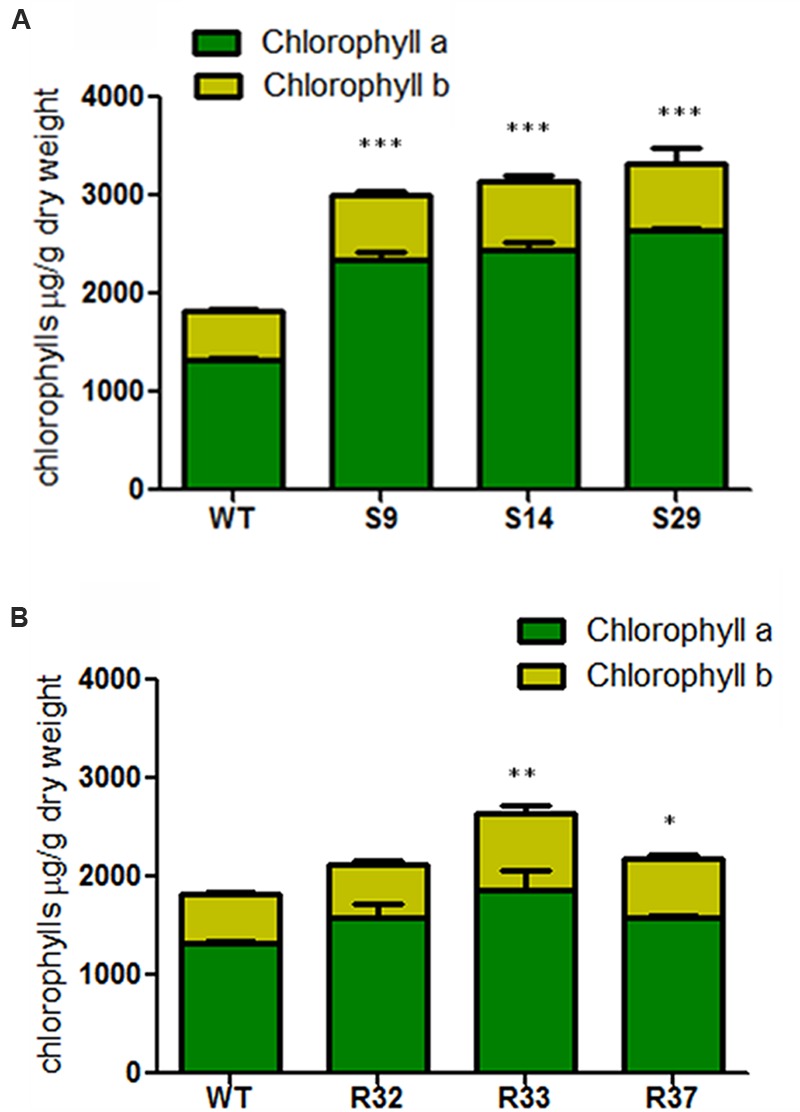
**Chlorophyll composition in leaves of carrot transgenic lines expressing *AtDXS* and *AtDXR*. (A)** Quantification of chlorophyll *a* and chlorophyll *b* in leaves of three DXS transgenic lines, **(B)** Quantification of chlorophyll *a* and chlorophyll *b* in leaves of three DXR transgenic lines. The sum of both chlorophyll *a* and chlorophyll *b* represents the total chlorophylls in wild-type (WT), and transgenic lines, Asterisks indicate significant differences between transgenic lines and wt determined two-tailed unpaired Student’s *t*-test. ^∗^*p* < 0.05; ^∗∗^*p* < 0.01; ^∗∗∗^*p* < 0.001.

Similar results were found for chlorophylls. Thus, the constitutive overexpression of *DXS* significantly increased the accumulation of chlorophylls in the leaves of all transgenic lines tested (**Figure 5A**). In particular, the levels of chlorophyll *a* in the best performing line (S29) were about twofold higher compared to the WT, whereas a 1.4-fold rise in chlorophyll *b* levels was detected in the same line (**Figure 5A**). The overexpression of *DXR* resulted in only minor changes in chlorophyll levels. While chlorophyll *a* did not change in any of the lines tested, modest but significant increases in the concentration of chlorophyll *b* and total chlorophylls were detected in some lines (**Figure 5B**). On average, increasing DXS activity led to 78% more chlorophyll *a*, 40% more chlorophyll *b* and 63% more total chlorophylls in carrot leaves, while increasing DXR activity did not change the levels of chlorophyll *a* but produced 38% more chlorophyll *b* and 21% more total chlorophylls compared to untransformed leaves (**Figure 5**).

### The Constitutive Overexpression of *Arabidopsis DXS* Increases the Transcript Abundance of PSY-Encoding Carrot Genes in Leaves

**FIGURE 6:**
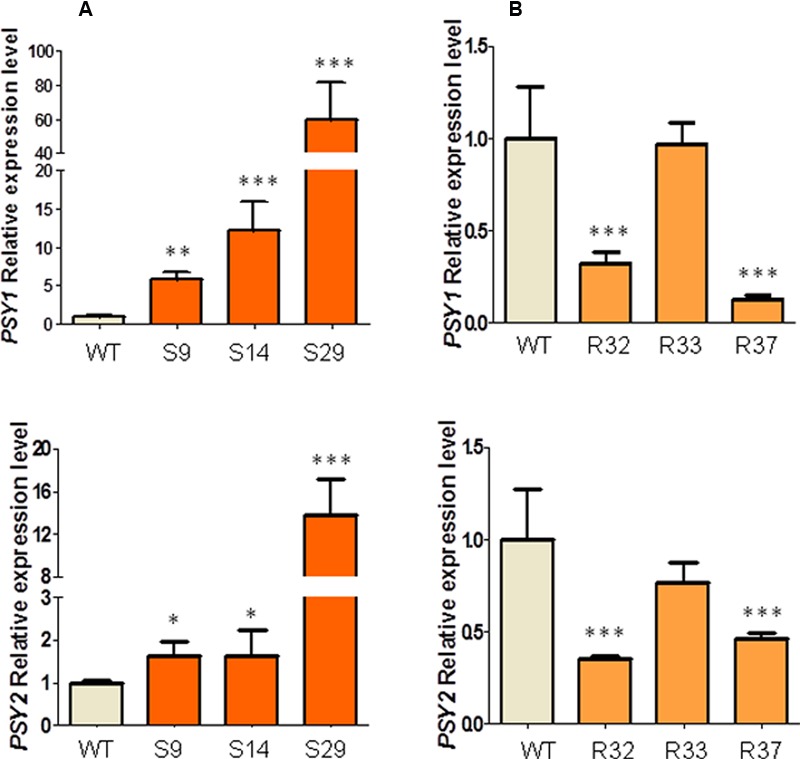
**Relative transcript expression of *DcPSY1* and *DcPSY2* in leaves of *AtDXS* and *AtDXR* carrot transgenic lines. (A)** *DcPSY1* and *DcPSY2* expression in leaves of three representative DXS transgenic lines. **(B)** *DcPSY1* and *DcPSY2* expression in leaves of three representative DXR transgenic lines. Assay was carried out in triplicate and normalized to *RNAr18S* expression. Expression of WT plants were used as calibrator and settled in 1. Asterisks indicate significant differences between transgenic lines and wt plants determined by two-tailed unpaired Student’s *t*-test. ^∗^*p* < 0.05; ^∗∗^*p* < 0.01; ^∗∗∗^*p* < 0.001.

While DXS has been shown to be the main rate-determining enzyme of the MEP pathway, PSY plays a similar role in the carotenoid pathway (Fraser et al., 2002; Wright et al., 2014). In fact, an increased DXS activity would only result in increased carotenoid biosynthesis if PSY activity is high enough to channel the extra supply of MEP-derived precursors to the carotenoid pathway. Consistent with the central role of these two enzymes in the control of the carotenoid pathway flux, a recent genome-scale analysis (Iorizzo et al., 2016) showed that carrot genes encoding DXS (DCAR_030576, named *DXS1*) and PSY (DCAR_023043 and DCAR_010057, respectively, named *PSY1* and *PSY2*) were differentially expressed in storage roots from populations with different carotenoid levels, showing increased transcript levels in accessions with higher carotenoid levels (Supplementary Figure S4). By contrast, no changes were found for the only carrot gene encoding DXR (DCAR_026133). Because transgene-mediated alterations of the carrot carotenoid pathway have been associated with a concomitant alteration of the transcript levels of endogenous *PSY1* and *PSY2* genes (Moreno et al., 2013), we next analyzed whether the transcript abundance of these two PSY-encoding genes was changed in leaves of representative lines overexpressing the *Arabidopsis DXS* and *DXR* genes. As shown in **Figure 6A**, leaves from transgenic plants overexpressing DXS showed a dramatic increase in the relative abundance of *PSY1* transcripts compared to WT controls. A lower increase was observed for *PSY2* transcripts (**Figure 6A**). In general terms, a fairly good correlation was found between levels of exogenous *DXS* transcripts (**Figure 2**) and endogenous PSY-encoding gene expression (**Figure 6A**) in leaves. Unexpectedly, however, we observed that the constitutive overexpression of *DXR* did not result in higher but lower levels of endogenous *PSY1* and *PSY2* transcripts in leaves from lines R32 and R37 and no changes in line R33 (**Figure 6B**).

## Discussion

Different studies, including metabolic control analysis, have shown that DXS is the main rate-determining enzyme of the MEP pathway (Wright et al., 2014; Rodriguez-Concepcion and Boronat, 2015). Consistently, a higher production of MEP-derived isoprenoids has been associated with increased expression of DXS-encoding genes in many plant systems. As indicated above, the carrot genome contains four genes for DXS-like sequences but only the one encoding the type I DXS isoform (*DXS1*) is expressed in correlation with the carotenoid content of carrot accessions displaying differentially pigmented roots (Iorizzo et al., 2016). While this observation suggested that DXS activity might regulate root carotenoid content, no experimental evidence was provided. Here show that increasing the levels of DXS, but not DXR, indeed results in an enhanced accumulation of carotenoids in dark-grown storage roots of transgenic carrot plants (**Figure 3**).

Unlike that described for roots, an upregulation of DXR levels in carrot leaves resulted in a modest increase in the total content of carotenoids (**Figure 4**) but also of other MEP-derived isoprenoids such as chlorophylls (**Figure 5**). A number of overexpression studies (Mahmoud and Croteau, 2001; Carretero-Paulet et al., 2006; Hasunuma et al., 2008; Yang et al., 2012; Chang et al., 2014; Zhang et al., 2015) support the conclusion that DXR contributes to the control of the MEP pathway flux but typically to a lower extent compared to DXS (Cordoba et al., 2009; Rodriguez-Concepcion, 2010). The increase in the levels of carotenoids and chlorophylls in the leaves of some of our transgenic DXR-overexpressing lines, however, was not a general effect. Moreover, the lack of correlation between transgene expression levels (**Figure 2**) and the accumulation of carotenoids (**Figure 4**) and chlorophylls (**Figure 5**) in transgenic lines suggests that the observed changes of isoprenoid pigment content might not be the direct consequence of altering DXR levels. Our results parallel similar observations in DXR-overexpressing spike lavender (*Lavandula latifolia*) plants (Mendoza-Poudereux et al., 2014). Together, we conclude that DXS plays a central regulatory function for the production of MEP-derived precursors in most plant systems, including carrot leaves and dark-grown roots, whereas the rate-determining activity of DXR appears not to be a general trend.

Our work further provides compelling evidence that the steps catalyzed by DXS and PSY enzymes represent regulatory nodes that coordinate the MEP pathway and the carotenoid pathway to ensure that the isoprenoid precursors required for carotenoid biosynthesis will be supplied when needed (Lois et al., 2000; Rodriguez-Concepcion et al., 2001; Fraser et al., 2007; Cordoba et al., 2009; Rodriguez-Concepcion, 2010). Increased PSY activity has been found to promote a post-transcriptional accumulation of DXS enzymes in Arabidopsis (Guevara-Garcia et al., 2005; Rodriguez-Villalon et al., 2009b) and DXS activity in tomato (Fraser et al., 2007). A possible mechanism involving the feedback regulation of DXS activity and turnover by IPP and DMAPP contents has been recently proposed to adjust the MEP pathway flux according to product (i.e., carotenoid) demand under normal growth conditions (Pokhilko et al., 2015). On the other hand, increased DXS activity in tomato fruits and potato tubers produces an increment in the expression of PSY-encoding genes (Lois et al., 2000; Morris et al., 2006) similar to that reported here in carrot leaves (**Figure 6**). Interestingly, a good correlation was found between the expression level of the DXS-encoding transgene (**Figure 2**) and the upregulation of carrot *PSY1* and *PSY2* genes (**Figure 6**) in individual lines, suggesting a direct effect. We speculate that the DXS-mediated induction of endogenous PSY-encoding genes in carrot might contribute to the increased accumulation of carotenoids detected in these lines. The increase in the production of chlorophylls in the leaves of DXS-overexpressing plants might be the consequence of increasing both the supply of MEP derived precursors and the carotenoid-mediated protection against photooxidative damage. However, it is also possible that chlorophyll biosynthetic genes could also be upregulated after increasing DXS activity. Further work should also determine whether other MEP-derived plastidial isoprenoids with roles in photosynthesis such as tocopherols and plastoquinone, and even hormones (**Figure 1**), are also altered in carrot leaves and roots with increased DXS (or DXR) activity. Because carotenoids (including those with provitamin A activity) are health-promoting metabolites and industrially relevant natural pigments (Fraser et al., 2007; Rodriguez-Concepcion, 2010), promoting their accumulation in plant-derived products by biotechnological methods will contribute to improve their economic and nutritional value

## Author Contibutions

KS: performed the experiments (carrot transformation, molecular analysis to select transgenic plants, HPLC quantification, qRT PCR) and wrote the manuscript, LQ: performed experiments (carrot transformation, molecular analysis to select transgenic plants). CS and MR-C: designed experiments and wrote the manuscript.

## Conflict of Interest Statement

The authors declare that the research was conducted in the absence of any commercial or financial relationships that could be construed as a potential conflict of interest.
