# Supplementary material for: Differential Contribution of the First Two Enzymes of the MEP Pathway to the Supply of Metabolic Precursors for Carotenoid and Chlorophyll Biosynthesis in Carrot (Daucus carota)
Source: Front Plant Sci. 2016 Aug 31;7:1344. doi: 10.3389/fpls.2016.01344 (PMC5005961; doi:10.3389/fpls.2016.01344)
Supplement: Supplementary file 1 [file Table_1.DOCX]

| **Gene** | **Secuence (5' - 3')** | **lenght (pb)** | **Description** |
| --- | --- | --- | --- |
| AtDXR F | AGTAGCGGATGCGTTGAAGC | 674 (AtDXR) and 1569 (AtDXS) | To check positive E.coli colonies and plants transformed with 35S:DXR:eGFP and 35S:DXS:eGFP. |
| AtDXS F | AGAGCTGGACTCGTTGGAGC |  |  |
| eGFP R | CGACCAGGATGGGCACCAC |  |  |
| qDXR F | CGAGTTGGTAACATCACCGTCTCTTG | 164 (qDXR) and 232 (qDXS) | To perform PCR and RT-PCR of AtDXR and AtDXS in combination with qeGFPR |
| qDXS F | CGCAGCAACCGCACTTAACTTAA |  |  |
| qeGFP F | GAACTTCAAGATCCGCCACAACAT | 121 | To perform RT-PCR of eGFP |
| qeGFP R | CAGGTAGTGGTTGTCGGG |  |  |
| 18S F | TTGATTACGTCCCTGCCCTTT | 196 | Housekeeping gene. |
| 18S R | ACAATGATCCTTCCGCAGGT |  |  |
| DcPSY1 F | AGTCGATGGAGCATTACCATAATTC | 95 | qRT PCR. DQ192186 |
| DcPSY1 R | CTAATGGGTTACAGAGGGTTGTGTTA |  |  |
| DcPSY2 F | GCTAATAAACTTCCGTGGGTGTTC | 112 | qRT PCR. DQ192187 |
| DcPSY2 R | GCTGGAGTTAGTGCTACCC |  |  |

**Table S1. List of primers used in this work**
