# Supplementary figures and images for: Differential Contribution of the First Two Enzymes of the MEP Pathway to the Supply of Metabolic Precursors for Carotenoid and Chlorophyll Biosynthesis in Carrot (Daucus carota)
Source: Front Plant Sci. 2016 Aug 31;7:1344. doi: 10.3389/fpls.2016.01344 (PMC5005961; doi:10.3389/fpls.2016.01344)

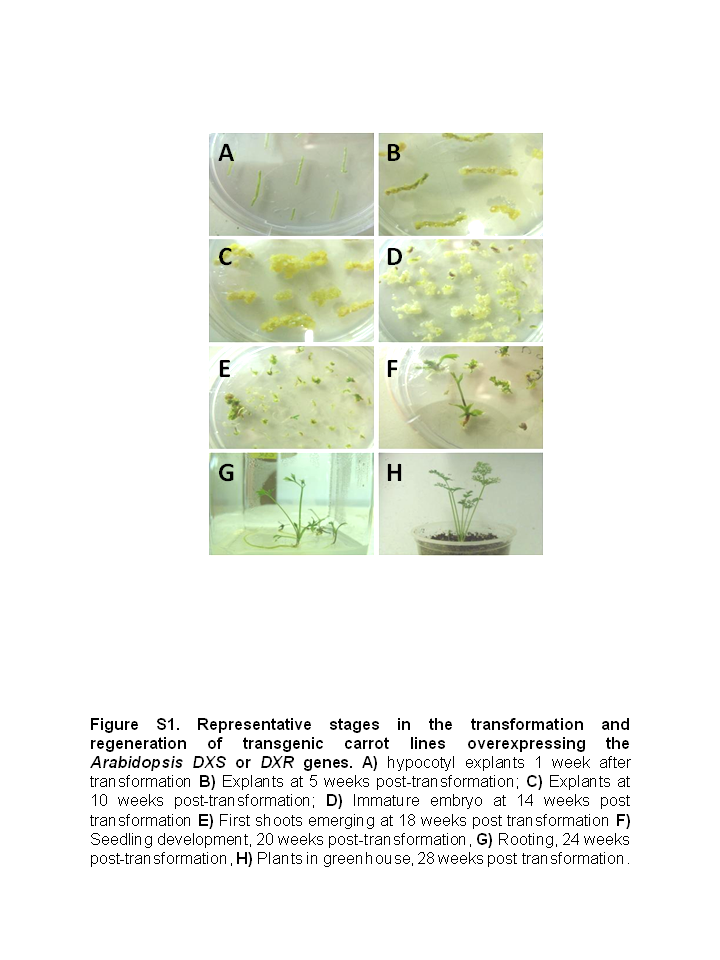

Supplement: Supplementary file 2 [file Image_1.TIF]

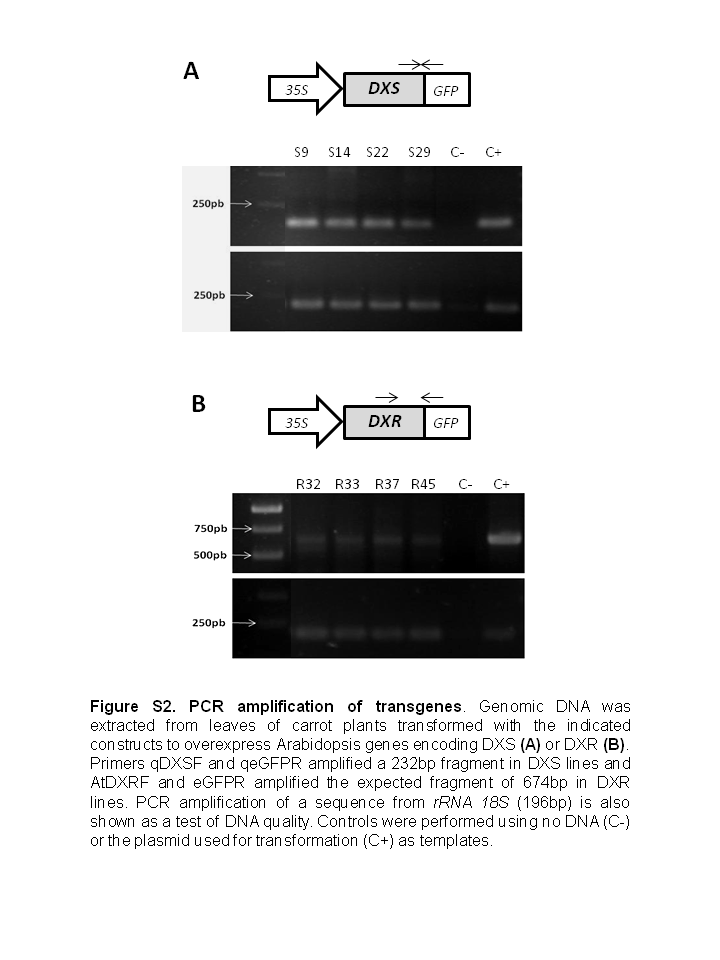

Supplement: Supplementary file 3 [file Image_2.TIF]

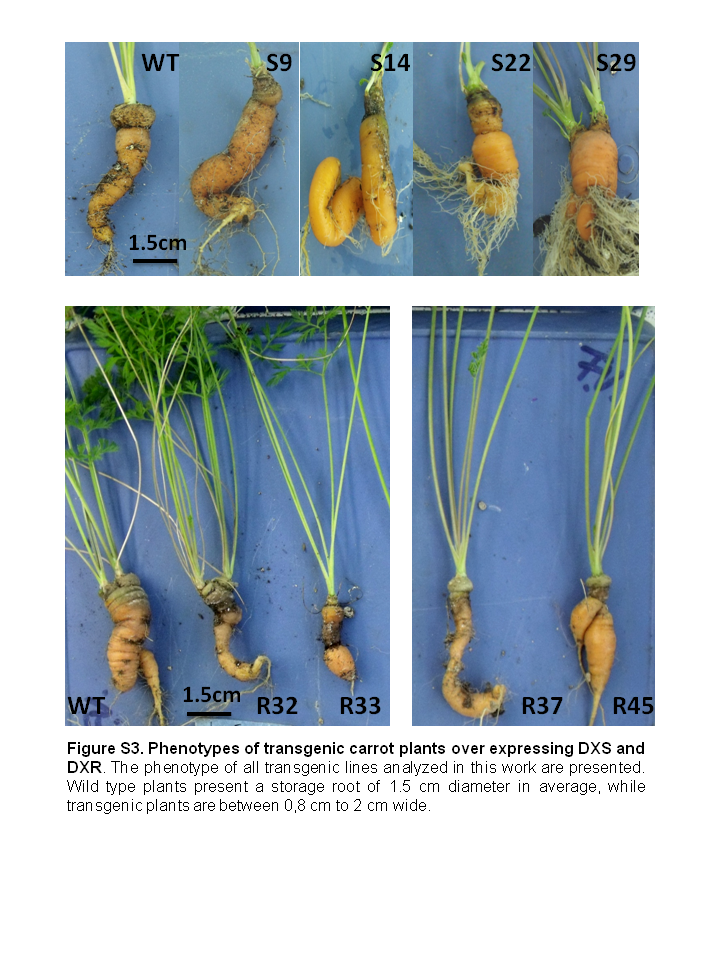

Supplement: Supplementary file 4 [file Image_3.TIF]

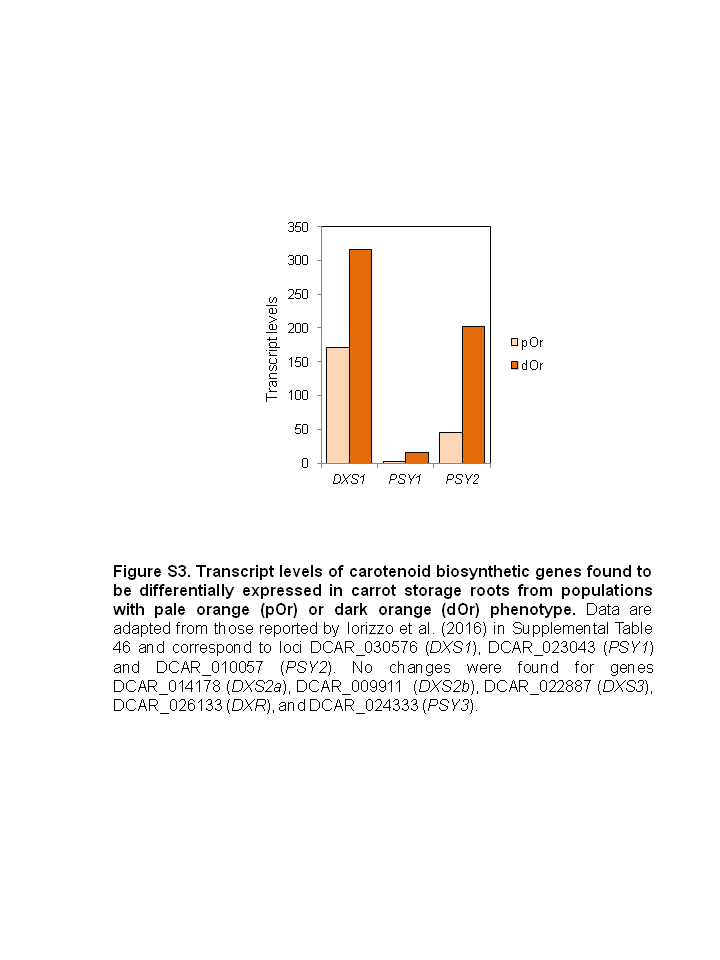

Supplement: Supplementary file 5 [file Image_4.TIF]
